# Arbidol: a broad-spectrum antiviral that inhibits acute and chronic HCV infection

**DOI:** 10.1186/1743-422X-3-56

**Published:** 2006-07-19

**Authors:** Yury S Boriskin, Eve-Isabelle Pécheur, Stephen J Polyak

**Affiliations:** 1 Departments of Laboratory Medicine, Microbiology and Pathobiology, University of Washington, Seattle, USA; 2 IFR128 Biosciences Lyon Gerland: Institut de Biologie et Chimie des Protéines, UMR 5086 CNRS-Université Claude Bernard Lyon I, Lyon, France; 3 Institute of Virology, Moscow, Russia

## Abstract

Arbidol (ARB) is an antiviral compound that was originally proven effective for treatment of influenza and several other respiratory viral infections. The broad spectrum of ARB anti-viral activity led us to evaluate its effect on hepatitis C virus (HCV) infection and replication in cell culture. Long-term ARB treatment of Huh7 cells chronically replicating a genomic length genotype 1b replicon resulted in sustained reduction of viral RNA and protein expression, and eventually cured HCV infected cells. Pre-treatment of human hepatoma Huh7.5.1 cells with 15 μM ARB for 24 to 48 hours inhibited acute infection with JFH-1 virus by up to 1000-fold. The inhibitory effect of ARB on HCV was not due to generalized cytotoxicity, nor to augmentation of IFN antiviral signaling pathways, but involved impaired virus-mediated membrane fusion. ARB's affinity for membranes may inhibit several aspects of the HCV lifecycle that are membrane-dependent.

## Background

There are presently limited therapeutic options for patients with chronic hepatitis C, especially those who have failed interferon (IFN) based modalities. The HCV replicon system, originally described by Lohmann and colleagues [1], has proven to be an effective *in vitro *model for pre-clinical evaluation and large-scale screening of new anti-HCV compounds (reviewed in [2]. In addition to the testing of novel anti-HCV compounds, the replicon system has also facilitated the characterization of existing compounds that show antiviral activity against other viruses [3,4]. Drugs that target viral proteins, such as the NS3-4a protease, are presumed to be less toxic and more specific. However, it is now clear that the use of these compounds can lead to drug-resistant viral variants, at least *in vitro *[5]. Another group of antivirals with broad-spectrum activity impact cellular metabolic pathways such as interferon production, or interfere with cellular functions or critical steps in virus-cell interactions, possibly exerting higher cell toxicity with little, if any, virus resistance. Other broad-spectrum antiviral agents target rate-limiting events in viral replication cycle such as envelope protein glycosylation, processing and folding [6], or viral-cell membrane fusion during viral uncoating or assembly (reviewed in [6,7]).

One example of the latter group compounds is arbidol (ARB; ethyl-6-bromo-4-[(dimethylamino)methyl]-5-hydroxy-1-methyl-2-[(phenylthio)methyl]-indole-3-carboxylate hydrochloride monohydrate), a Russian-made broad-spectrum antiviral that had been shown to inhibit the fusion of influenza A and B viruses within endosomes [8]. An acidic environment (pH 5.0) is a strict prerequisite for influenza virus-induced fusion, and even a slight increase in pH abolishes the fusion process [9]. ARB has also been shown to exert antiviral activity against other pH-dependent viruses, such as hepatitis B virus [10], and rhinovirus 14 (reviewed in [7]). Since ARB is a weak base, it might elevate endosomal pH and abrogate virus-endosome fusion. Since optimal HCV envelope protein fusion with cell membranes requires low pH and the fusion process may occur within endosomes [11-14], ARB might potentially exert an antiviral activity on HCV as well. However, since ARB was shown to inhibit various pH-independent viruses such as the human respiratory syncytial virus and the parainfluenza virus 3 [7], ARB could affect membrane interactions that are necessary for virus replication. Alternatively, ARB has been claimed to stimulate the production of interferon [7,8], a well-known and potent inhibitor of HCV replication [15]. Taken together, these observations prompted us to investigate ARB activity on HCV lifecycle, and more specifically on HCV replication. We therefore studied the effects of ARB on HCV RNA and protein expression using primary productive HCV infection or persistent non-productive HCV infection in cultured replicon cells. We report that ARB inhibits acute and chronic HCV replication independently of activation of innate antiviral signaling pathways. Instead, we show that ARB antiviral activity towards HCV is due to a direct effect of ARB on virus-cell membrane interactions.

## Results

### Effects of ARB on cell viability

**Figure 1:**
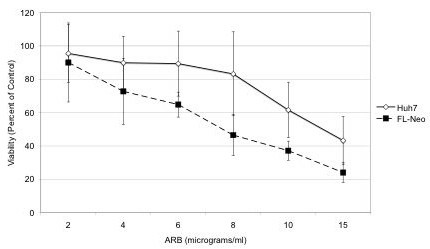
**Determination of CC50 for ARB**. FL-Neo or Huh7 cells were treated with increasing concentrations of ARB for 72 hours and assessed for viability using the ATPlite assay. Each point on the curve represents the average of six replicates.

Huh7, Huh7.5.1 or FL-NEO cells were treated with ARB concentrations in the range of 2 μg/ml (3.8 μM) to 15 μg/ml (28.2 μM). These doses of ARB were previously found to exert anti-viral effects (Brooks et al., 2004). Cell viability was measured by ATP fluorescence. As shown in Figure 1, the concentration of ARB that reduced FL-Neo and Huh7 viability by 50% (CC50) was 8.0 and 12.5 μg/ml, respectively. FL-Neo cells were slightly more sensitive to the effects of ARB, an effect that could be due to increased cell stress as a result of high level expression and replication of the entire HCV genome. The CC50 for Huh7.5.1 cells was similar to Huh7 cells (data not shown). From these observations, we treated FL-Neo replicon cells with 6 μg/ml ARB and Huh7 and Huh7.5.1 cells with 8 μg/ml ARB in subsequent experiments.

### ARB inhibits chronic HCV replication

**Figure 2:**
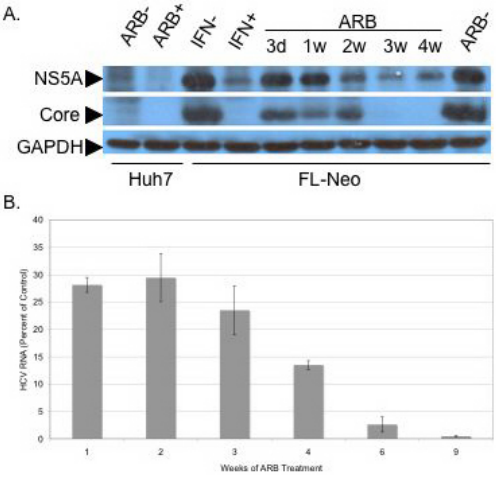
**Arbidol inhibits chronic HCV replication**. FL-Neo replicon cells were passaged without G418 in the presence of 6 μg/ml (11 μM) ARB for 3 days (lane 3d), or 1, 2, 3 or 4 weeks (lanes 1w to 4w). Huh7 cells, as a control for non HCV-replicating cells, were treated (lane ARB +) or not (lane ARB -) at 6 μg/ml for 3 days. The rightmost lane labeled ARB- represents proteins from control FL-Neo cells, treated without ARB for 4 weeks. FL-Neo cells were separately treated with 100 U/ml IFN-α for 48 h (lane IFN +) or not (lane IFN -). Cells were then lysed and treated as described in Materials and Methods. Ten μg of total cell protein of each sample extract were submitted to SDS-PAGE, followed by western blotting with murine monoclonal antibodies to NS5A or core proteins, or polyclonal antiserum to GAPDH.

FL-Neo cells were cultured in the presence of 6 μg/ml of ARB from 3 days to several weeks, with weekly analysis of viral proteins expression by Western blot. Cells were fed with ARB daily since its half-life in cultured cells is about 18 hours [16]. At this concentration, cells did not appear to be stressed, nor did the cells display significant cytotoxicity during regular cell passage and trypan blue counting. Figure 2A presents HCV core and NS5A protein expression data and shows that the anti-viral effect of ARB appeared during the second week of treatment. HCV core protein became virtually undetectable after 3 weeks of ARB treatment onwards. The level of HCV NS5A and core protein expression after 3 weeks of ARB treatment was similar to the inhibition of viral proteins following 48 hours of IFN-α treatment. HCV RNA levels in ARB-treated cells also gradually declined (Figure 2B) until they dropped to 0.45% of that of untreated FL-Neo cells on the ninth week post-treatment. After 10 weeks of ARB treatment, cells were split in two halves: one was left in Huh7 medium (lacking G418) with ARB and the other transferred to G418-supplemented medium lacking ARB. In the presence of G-418, FL-Neo cells displayed extensive cytopathic effect. After two more weeks of culture, G-418-treated FL-Neo cells perished, presumably due to the loss of replicon RNA and the concomitant loss of neomycin resistance. The other half of ARB-treated, G418-free culture was extracted for RNA and subjected to real time RT-PCR. HCV RNA was undetectable by real time RT-PCR (data not shown), indicating that after 10 weeks of ARB treatment the FL-Neo cells were cured of HCV infection, with no HCV rebound in the following two weeks (data not shown).

### ARB inhibits acute HCV infection

**Figure 3:**
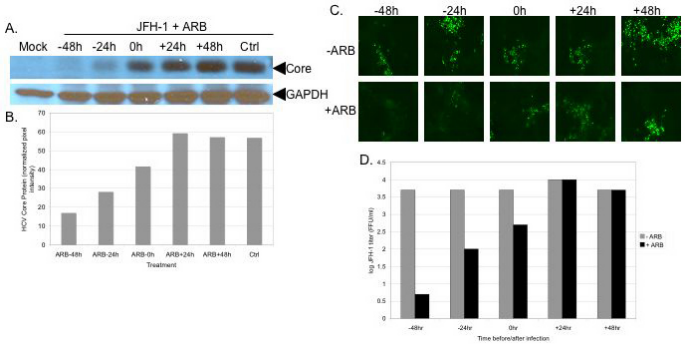
**ARB inhibits acute HCV infection**. A: HCV core protein inhibition in ARB-pretreated Huh7.5.1 cells. Cells were pre-incubated with 8 μg/ml ARB for 24 (-24 h) or 48 (-48 h) hours prior to infection with JFH-1 (moi 0.02) for 72 hours. Cells were also treated with ARB at the time of JFH-1 infection (0 h), and treated 24 (+24 h) and 48 (+48 h) hours post-infection. Equal amounts of total protein were probed for HCV core with a monoclonal antibody. Blots were stripped and reprobed for GAPDH expression to verify equal loading of protein in all lanes. B: quantitation of HCV core protein expression. Blots in panel A were scanned and pixel intensity measured using Image J software. Data were normalized to GAPDH levels. C: Suppression of infectious JFH-1 yields in ARB-pretreated cells. Cell-free supernatants from the experiment described in panel A were quantitated for infectious virus by the focus-forming assay as described in the Materials and Methods. Green foci depict NS5A expressing cells. D: Quantitation of JFH-1 titers from panel C. Supernatants from control and ARB treated cells were serially diluted and the titer of virus determined using the focus-forming assay. Titers were calculated by counting the number of foci and correcting for the dilution factor, as described previously [17].

Huh7.5.1 cells were seeded in 6-well plates at 0.5 × 10^6 ^cells/well and infected the next day with a JFH-1 viral stock at a multiplicity of infection of 0.02. ARB (8 μg/ml) was added to cells 48 hours (-48 h) or 24 hours (-24 h) before infection, at the time of infection (0 hrs) and 24 hours (+24 h) or 48 hours (+48 h) post-infection. At 72 hours post-infection the extracellular virus was collected for infectious virus titration, and cells were lysed for Western blot analysis of HCV core protein expression. The results of protein analysis showed that ARB had the most pronounced inhibitory effect in pre-treated cells (24 and 48 hrs before infection). Minimal anti-HCV effects were observed if ARB was added at the time of infection. ARB did not exert any antiviral effects when it was added 24 or 48 hrs post-infection (Figure 3A and 3B). These results were in agreement with yields of infectious JFH-1 virus, measured via immunofluorescent detection of the HCV NS5A protein by a focus forming assay [17] (Figure 3C). Figure 3D demonstrates that ARB inhibited infectious JFH-1 virus production up to a 1000-fold when given to cells 48 hours prior to infection, and a 100-fold HCV titer reduction when the drug was given 24 hrs before infection. Addition of ARB at the same time or 24–48 hours after JFH-1 infection did not significantly affect virus yields (Figure 3C and 3D). The data indicate that ARB inhibits acute HCV infection when administered prophylactically.

### ARB does not affect RIG-I and IFN signaling pathways

**Figure 4:**
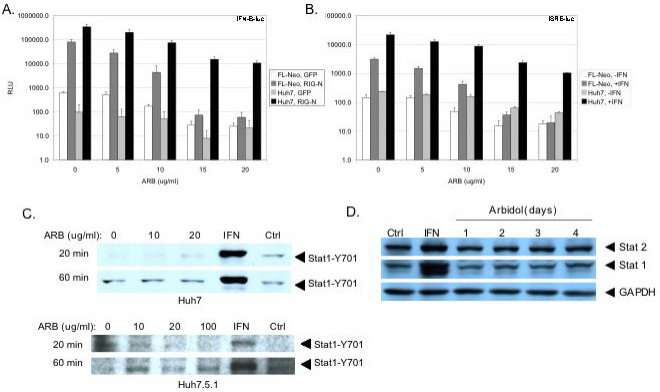
**ARB does not induce IFN antiviral responses**. A, ARB does not induce RIG-I dependent signaling. FL-Neo replicon and Huh7 cells were co-transfected with IFNB-luciferase and GFP or IFNB-luciferase and constitively active RIG-N expressing plasmids for 3 hours. Transfection mixtures were then removed and cells were incubated with medium containing the indicated concentrations of ARB. Luciferase activity was measured 24 hours later. B, ARB does not induce ISRE transcription. FL-Neo and Huh7 cells were transfected with ISRE-luciferase reporter plasmids for 3 hours. Transfection mixtures were then removed and cells were incubated with medium containing the indicated concentrations of ARB for 20 hours. Cells were then treated or not treated with 100 U/ml of IFN-α (Roferon) for 4 hours before luciferase activity was measured. C, ARB does not induce Stat1 phosphorylation on the conserved tyrosine amino acid at position 701. Huh7 or Huh7.5.1 cells were treated with the indicated amounts of ARB and whole cell protein extracts were harvested 20 and 60 minutes later. Cells were also treated with 500 units per milliliter of IFN-α, or as a negative control for possible solvent effects, an equivalent volume of ethanol (Ctrl). The position of Stat1-Y01 is indicated with arrows. D, ARB does not induce IFN stimulated gene expression. FL-Neo cells were treated with 6 μg/ml ARB for 1 to 4 days, or treated with 100 U/ml IFN-α for 24 hours. Whole cell protein extracts were probed for Stat1, Stat2, and GAPDH protein levels.

Since IFN is known to inhibit HCV replication, we determined the effects of ARB on innate antiviral signal transduction pathways in FL-Neo and Huh7 cells. We measured activation of the IFN-β promoter by retinoic acid inducible gene 1 (RIG-I), a key factor in double stranded RNA signaling in response to HCV infection [18]. We also measured basal and IFN-α induced ISRE transcription, a measure activation of Jak-Stat pathway activation via the ISGF-3 transcription factor, which is a complex of Stat1-Stat2-IRF9 proteins [19]. ISRE activation occurs downstream of IFN-β activation during virus infection [20]. As shown in Figure 4A, transfection of FL-Neo replicon and Huh7 cells with RIG-N, a constitutively active mutant of RIG-I [21], caused robust induction of IFN-β transcription, as compared to cells that expressed control green fluorescent protein (GFP). Addition of ARB to cells did not modify the basal level of RIG-N-induced IFN-β transcription. Rather, ARB caused a dose-dependent inhibition of IFN-β transcription in all conditions. Figure 4B demonstrates that IFN-α treatment of FL-Neo and Huh7 cells activates ISRE transcription, and that ARB dose dependently inhibits basal and IFN-induced ISRE promoter activity. Moreover, treatment of Huh7 and Huh7.5.1 cells with ARB did not induce phosphorylation on the conserved tyrosine amino acid at position 701 of the Stat1 protein (Figure 4C), an essential requirement and indicator of IFN signaling through the Jak-Stat pathway [19]. As a control, IFN treatment of cells for 20 or 60 minutes induced robust Stat1-Y701 phosphorylation in both cell types. Finally, incubation of FL-Neo cells with ARB for up to 4 days did not increase the expression of the ISGs Stat1 and Stat2 (Figure 4D). In contrast, treatment of cells with IFN-α for 24 hours induced Stat1 and Stat2 proteins. Collectively, the data indicate that ARB does not induce an IFN antiviral response in hepatocyte cultures that could account for the inhibition of HCV replication by ARB.

### ARB inhibits HCV membrane fusion

**Figure 5:**
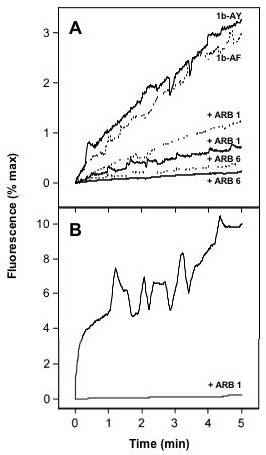
**Arbidol inhibits HCVpp-mediated lipid mixing**. Lipid mixing curves of HCVpp genotype 1b (panel A) and of HApp (panel B) in the absence or presence of ARB, with R_18_-labeled liposomes (representative of 3 separate experiments). Fourty μl of HCVpp-1b (panel A, solid lines represent 1b-AY for Genbank accession number AY734975, while dotted lines are labeled as 1b-AF for Genbank accession number AF333324) or HApp (panel B) were added to R_18_-labeled phosphatidylcholine:cholesterol liposomes (15 μM final lipid concentration), in PBS pH 7.4 at 37°C, with or without 1 (ARB 1) or 6 μg/ml (ARB 6) ARB. After a 2-min equilibration, lipid mixing was initiated by decreasing the pH to 5.0 (time 0), and recorded as R_18 _fluorescence dequenching as a function of time. The 100% fluorescence was obtained by adding 0.1% (v:v; final concentration) Triton X-100 to the suspension.

As mentioned earlier, ARB exerts its antiviral activity on influenza virus partly through the inhibition of viral membrane fusion in the endosome. Since the optimal pH for HCV fusion is at 5.5 [13,14], we reasoned that ARB might similarly affect HCV fusion. This was tested in our recently developed *in vitro *fusion assay using fluorescently-labeled liposomes and HCV pseudoparticles (HCVpp) harboring HCV E1/E2 glycoproteins at their surface, and assembled around a retroviral core [14,22]. As shown in Figure 5A, ARB displayed a dose-dependent inhibition of HCVpp-mediated lipid mixing, with fusion becoming virtually undetectable at a concentration 1 μg/ml ARB. This was observed for HCVpp derived from two different HCV genotype 1b isolates (AY734975 and AF333324). As a positive control, ARB also completely inhibited the fusion of pseudoparticles bearing the influenza virus hemagglutinin (HA) protein (Fig. 5B). The inhibition was observed immediately after addition of ARB to the cuvette, and was similar when either HCVpp-1b or liposomes were pre-incubated with the drug. Moreover, since our fusion assay is based upon the use of plain lipid vesicles, which do not contain cell surface receptors for HCV, the inhibition is probably not due to a direct effect of ARB on the HCV glycoproteins E1 and/or E2. Rather, the data suggest that the effect of ARB on virus/liposome interactions occurs at the membrane level. This is strengthened by the observation that ARB exerts a similar fusion inhibiting effect on HCVpp harboring glycoproteins from other genotypes as well (Pécheur EI, Boriskin Y, Lavillette D, Roberts M, Cosset FL, Polyak SJ, manuscript in preparation). Taken together, the data suggest that ARB interacts with membranes and likely perturbs stages of the HCV lifecycle that are membrane-dependent.

## Discussion

The advent of HCV replicon cultures [1,23] and in particular, productive replicon infection systems [17,24,25], has given tremendous opportunities for preclinical assessment of anti-HCV compounds. As a result, a number of specific viral inhibitors are already in various phases of clinical trials (reviewed in [5]). Among the non-specific, broad-spectrum antivirals, a few well-known over-the-counter drugs have shown inhibitory activity against HCV in replicon cell cultures [3,4]. This latter group of antivirals can now be extended to include ARB, which exerts anti-viral activity against acute and chronic HCV replication.

Based on its chemical structure, ARB may be a pro-drug which becomes chemically converted into an active drug by cellular metabolic processes. The pro-drug nature of ARB may explain its relatively high CC50 values, assuming that the actual ARB metabolite concentration is lower than that of the ARB pro-drug. The carboxylic acid ester moiety contained in the structure of ARB may be a substrate for hydrolysis in vivo that leads to the intracellular accumulation of ARB. The fact that ARB displayed prophylactic activity when administered 24–48 hours before primary infection, and over several weeks of treatment in persistent HCV infection, might indicate a prerequisite for ARB accumulation in intracellular compartments before antiviral activity is observed. Clearly, additional studies of ARB and various chemical derivatives are warranted. Nonetheless, the sustained effect of ARB on persistent HCV could be of clinical significance since chronic infection comprises about 75–85% of all hepatitis C cases [26].

Our results suggest that the inhibitory effect of ARB on HCV is not mediated by stimulation of type 1 IFN signaling pathways. In fact, ARB inhibited antiviral signaling in FL-Neo and Huh7 cells, an effect which might be attributable to ARB disruption of membrane interactions required for signal transduction. Instead, ARB's anti-HCV action appears to be attributable to an inhibitory effect on viral fusion (Pécheur EI, Boriskin Y, Lavillette D, Roberts, M, Cosset FL, Polyak SJ, manuscript in preparation). In brief, at 1–6 μM concentration, ARB completely blocked the fusion of HCV pseudoparticles from multiple genotypes with liposomal membranes in a strictly controlled pH environment of 5.0. This observation makes it unlikely that ARB inhibits virus replication by increasing the endosomal pH like other weak bases such as chloroquine [27]. Moreover, since HCV fusion takes place within much wider range (6.3 – 5.0) compared to strictly HA conformation-sensitive influenza virus fusion [14], if ARB did induce a basic pH shift, it is predicted that the shift would be minor and affect the HCV fusion process minimally. One model for ARB's anti-fusion activity is that ARB, because of its chemical structure, has a propensity for cell membranes. If ARB intercalates into membranes and adopts a consistent orientation, the formation of an "ARB cage" could lead to excessive stabilization of cell membranes, and thereby prevent HCV fusion. Alternatively, at the virus level, ARB might block the un-coating of the membrane during the fusion process. It is also conceivable that ARB could inhibit other aspects of the HCV life cycle that are dependent on cell membranes. For example, the HCV replication complex associates with endoplasmic reticulum membranes to form membranous webs [28]. The web is formed via the association of HCV non-structural proteins with ER membranes [29]. Thus, ARB-induced inhibition of HCV non-structural protein interactions with organelle membranes might also contribute to suppression of HCV replication. However, at least in terms of acute infection with JFH-1, the predominant mechanism of action of ARB is likely to be inhibition of fusion, since ARB only suppressed JFH-1 infection when given 24–48 hours prior to infection. On the other hand, FL-Neo cells were cured of HCV replication following several weeks of ARB treatment. Since genomic length replicons do not produce infectious virus, in these cells, ARB inhibition of the HCV replication complex association with membranes could be the predominant mode of HCV suppression. Additional studies are required to sort out these possibilities.

In conclusion, ARB inhibits HCV acute and chronic HCV infection and replication. The inhibitory effect appears to be due to the interaction of ARB with membranes and to subsequent ARB-induced membrane alterations, but the nature of the membrane modification(s) require further study.

## Materials and methods

### Cells, culture media, drug preparation, plasmids

Huh7 and Huh7.5.1 human hepatoma cells were cultured in Dulbecco's modified Eagle medium (DMEM) containing 9% fetal calf bovine serum, 1% penicillin-streptomycin-fungizone and 1% nonessential amino acids (all reagents were from Invitrogen, Carlsbad, CA). FL-Neo cells are a stable human hepatoma Huh7-derived cell line harboring autonomously replicating genomic length genotype 1b HCV replicon with adaptive mutations in NS3 (P1496L) and NS5A (S2204I). Huh7 and FL-Neo cells were obtained from Apath, LLC (St Louis, MO), and Huh7.5.1 cells were obtained from Francis Chisari [17]. FL-Neo cells were cultured in Huh7 medium supplemented with 0.4 mg/ml of G418 (Calbiochem, San Diego, CA). They were passaged with a 1:10 split and maintained subconfluent in the course of each experiment. During short- or long-term ARB treatment, FL-Neo cells were cultured in medium without G418. Control cultures consisting of FL-Neo cells grown in the absence of ARB were always run in parallel with ARB treated cultures, and for the same duration. All cell lines were checked for mycoplasma using MycoAlert assay (Cambrex Bio Science, Rockland, ME) and found to be mycoplasma-free.

ARB was a powdered free base formulation and was dissolved to completion in 0.5 ml of 96-proof ethanol at 37°C for 10 min followed by dilution in 4.5 ml of sterile distilled water. The final ethanol content in cell culture medium was always less than 10^-6 ^M. For each experiment a freshly prepared stock of ARB was used. JFH-1 viral stock preparation, cell infection and titration was performed exactly as described [17,25]. JFH-1 plasmid was kindly provided by Takashi Wakita. RIG-N, a constitutively active mutant of RIG-I, was kindly provided by Michael Gale.

### Cellular toxicity assay

We used the luminescence ATP detection assay system (ATPlite, Perkin Elmer, Boston, MA) as described by the manufacturer. Huh7, Huh7.5.1 or FL-Neo cells were grown overnight in black 96-well view plates (10^4 ^cells per well, 6 wells per drug concentration). After 24 h incubation with the compound the wells were washed twice with 0.2 ml of phosphate-buffered saline (PBS) followed by addition of 0.1 ml of PBS and 50 μl of lysis solution (provided with the kit) to each well. The microplate was shaken for 5 min at 600 rpm on an orbital shaker to allow cell lysis and ATP stabilization. 50 μl of the substrate solution was then added, and the microplate was shaken for 5 min at 600 rpm. Luminescence was measured on a TopCount NXT microplate scintillation & luminescence counter (Packard; Perkin Elmer) after a 10 min dark adaptation. The 50% cytotoxic concentration (CC50) was determined from the dose-response curve and defined as the drug concentration that caused a 50% signal reduction compared to that of untreated cultures.

### Western blotting

Cells grown in Costar 6-well plates (0.2 × 10^6 ^cells per well) were lysed in 0.1 ml of RIPA buffer (50 mM tris-HCl, pH 7.2, 150 mM NaCl, 0.1% SDS, 0.1% Na deoxycholate, 1% Triton X-100, 17.4 μg/ml PMSF). The protein lysates were quantified using the BSA Protein Assay (Pierce Biotechnology, Rockford, IL). Before gel loading each sample was adjusted to contain 10 μg of protein per gel well. Samples were mixed with equal volume of double-strength reducing loading buffer and heated at 95°C for 7 min before subjected to electrophoresis on a 4–20% tris-glycine gel (Invitrogen). Separated proteins were transferred to a 0.45 μm nitrocellulose membrane (Pierce) using a semi-dry transfer system. After the transfer, the membrane was blocked in Superblock buffer (Pierce) and incubated with primary mouse antibody, either anti-NS5A (Biodesign International, Saco, ME.; 1:1000 dilution) or anti-Core (Affinity Bioreagents, Golden, CO; 1:1000 dilution) for 1 hr at room temperature. Both proteins were detected on the same blot after sequential treatment with anti-NS5A, then with anti-Core antibody, and four washes with PBS – 0.2% Tween 20 (PBST) in between antibody treatment. The secondary antibody was HRP-conjugated anti-mouse immunoglobulin G (IgG) (Pierce, 1:10000 dilution). To control for comparable gel loading, the same blot was stripped with Restore stripping buffer (Pierce), and was re-blocked in Pierce Superblock buffer and incubated overnight with goat polyclonal IgG antibody to glyceraldehyde-3-phosphate dehydrogenase (GAPDH; Santa Cruz Biotechnology, Santa Cruz, CA) diluted 1:1000 in PBST. The blot was washed as above and incubated with bovine anti-goat IgG-HRP (Santa Cruz Biotechnology) diluted 1:10000 in PBST, for 1 hr at room temperature. Protein bands were detected using chemiluminescence LumiGlo reagents (Cell Signaling, Danvers, MA) and visualized after exposing the membrane against X-ray film. Quantification of bands was performed using ImageJ software [30].

### HCV RNA quantitation

Huh7 or FL-Neo cells were seeded in Costar 6-well clusters at 2.0 × 10^5 ^cells per well. Sub-confluent treated or untreated cultures were lysed in 0.6 ml/well of RLT buffer (Qiagen) containing 1% beta-mercaptoethanol. Total cellular RNA was isolated using the RNeasy kit (Qiagen, Valencia, CA) according to manufacturer's instructions. The RNA integrity was verified by visualizing ribosomal RNAs on 1.2% agarose gel, and total RNA concentration was determined using RediPlate™ 96 Ribogreen RNA quantitation Kit (Molecular Probes, Invitrogen). Ten ng of RNA were added to wells of a 384 well plate containing the EZ RT-PCR master mix (Perkin Elmer). Samples were run on an ABI HT7900 real time RT-PCR machine. HCV RNA was quantitated by real time RT-PCR, as described [31,32]. For each run, dilutions of HCV plasmid DNA (precisely quantitated using Invitrogen PicoGreen DNA quantitation kit) ranging from 0–10^7 ^copies per tube, were run in triplicate to generate a standard curve, which served as a reference to calculate HCV RNA copy number based on the cycle threshold (Ct). The HCV RNA copy number was expressed as copies per 10 ng total cellular RNA. Negative controls included reactions lacking template as well as RNA from Huh7 cells, which were always negative for HCV RNA.

### HCV pseudoparticle (HCVpp) assay

The HCV pseusoparticle (HCVpp) system was described in details elsewhere [14,22]. The measure of fusion between pseudoparticles and liposomes was based upon a lipid mixing assay, as described earlier [14]. Briefly, liposomes of phosphatidylcholine/cholesterol labeled with octadecylrhodamine (R_18_) (65:30:5 mol/mol) were incubated at 37°C with HCVpp harboring the E1 and E2 glycoproteins of HCV genotype 1b isolates AY734975 [33] and AF333324, in the absence or presence of 1 or 6 μg/ml ARB. Lipid mixing was initiated by decreasing the pH to 5.0 with diluted HCl, and kinetics were recorded over a 10-min period of time on an SLM Aminco 8000 spectrofluorimeter, at λ_exc _560 nm and λ_em _590 nm.

## Abbreviations

**ARB**: arbidol; **HA**: hemagglutinin; **HCV**: hepatitis C virus; **HCVpp**: HCV pseudoparticle; **IFN**: interferon;**IFNB**: IFN-beta;**ISG**: IFN-stimulated gene; **ISRE**: IFN stimulated response element; **JFH-1**: Japanese fulminant hepatitis-1;**NS3-4a**: non-structural 3-4a; **NS5A**: non-structural 5A; **RT-PCR**: reverse transcriptase polymerase chain reaction; **RIG-I**: retinoic acid inducible gene I;**SDS-PAGE**: sodium dodecyl sulphate-polyacrylamide gel electorphoresis; **Stat**: signal transducer and activator of transcription.
